# Effects of mitochondrial dysfunction on bone metabolism and related diseases: a scientometric study from 2003 to 2022

**DOI:** 10.1186/s12891-022-05911-8

**Published:** 2022-11-26

**Authors:** Wei Zhang, Chang-Liang Xia, Jun-Nan Ma, Jia-Xuan Li, Qi Chen, Shuan-Ji Ou, Yang Yang, Yong Qi, Chang-Peng Xu

**Affiliations:** 1 Department of Orthopaedics, Guangdong Second Provincial General Hospital, NO.466 Xingang Road, Haizhu District, Guangzhou, 510317 P.R. China; 2 The Second School of Clinical Medicine, Southern Medical University, Guangzhou, China; 3 Jinan University, Guangzhou, 501632 China

**Keywords:** Bone metabolism, Bone metabolic diseases, Osteoarthritis, Mitochondrial dysfunction

## Abstract

**Background:**

In recent years, mitochondrial dysfunction has been extensively studied and published, but research on the effects of mitochondrial dysfunction on bone metabolism and related diseases is only just beginning. Furthermore, no studies have been carried out to systematically illustrate this area from a scientometric point of view. The goal of this research is to review existing knowledge and identify new trends and possible hotspots in this area.

**Methods:**

All publications related to the relationship between mitochondrial dysfunction and bone metabolism and related diseases from 2003 to 2022 were searched at the Web of Science Core Collection (WoSCC) on May 7, 2022. Four different analytical tools: VOSviewer 1.6.18, CiteSpace V 6.1, HistorCite (12.03.07), and Excel 2021 were used for the scientometric research.

**Results:**

The final analysis included 555 valid records in total. Journal of Biological Chemistry (Co-citations = 916) is the most famous journal in this field. China (Percentage = 37%), the United States (Percentage = 24%), and Korea (Percentage = 12%) are the most productive countries. Blanco FJ and Choi EM are the main researchers with significant academic influence. Current research hotspots are basic research on mitochondrial dysfunction and the prevention or treatment of bone metabolism-related diseases.

**Conclusion:**

The study of the consequences of mitochondrial dysfunction on bone metabolism and associated diseases is advancing rapidly. Several prominent researchers have published extensive literature and are widely cited. Future research in this area will focus on oxidative stress, aging, gene expression, and the pathogenesis of bone metabolism-related diseases.

**Supplementary Information:**

The online version contains supplementary material available at 10.1186/s12891-022-05911-8.

## Introduction

Mitochondria are a specialized organelle found widely in eukaryotic cells, and they have circular DNA that can replicate independently [1]. The basic function of mitochondria is to act as the final oxidation site for sugars, lipids and amino acids, releasing energy for oxidative phosphorylation to synthesize ATP [2]. In addition, mitochondria play an important role in regulating calcium ion concentration, regulating cellular metabolism and signaling networks, and regulating cell expression and apoptosiss [3–6]. The important role of mitochondria in growth, development, metabolism, aging, disease, death, and biological evolution is receiving increasing attention. Studies have shown that many human diseases, such as tumors, type 2 diabetes, and neurodegenerative diseases are associated with varying degrees of mitochondrial dysfunction [7–10]. Bone is a highly mineralized organ with a constantly active metabolism in which osteoblasts, osteoclasts, chondrocytes, and bone marrow mesenchymal stem cells maintain a balance in bone matrix and mineral deposition and absorption by participating in and regulating different metabolic pathways [11, 12]. In fact, impairment of homeostasis and/or metabolic processes in the body can lead to various congenital bone and bone diseases [13–15].

The Scientometric analysis assesses the influence of particular research discoveries and the evolution of literature in certain fields, as well as evaluating scientific research trends [16, 17]. It analyzes the metrological features of the paper[18], identifying keywords for various characteristics such as countries, institutions, journals, authors, and papers in specific fields [17], giving researchers guidance and suggestions for future research by providing an overview of the current status and development trends in a specific field [19]. In general, there are three steps in doing scientometric analysis: 1) searching literature from searchable databases; 2) analyzing data using software tools; 3) preparing manuscripts for publication. The WoSCC is regarded as the most popular database for scientometric studies [17, 19, 20], and contemporary science metrology analysis software includes HistorCite [21], VOSViewer [22], and CiteSpace [23].

## Data source and search strategy

**Fig. 1:**
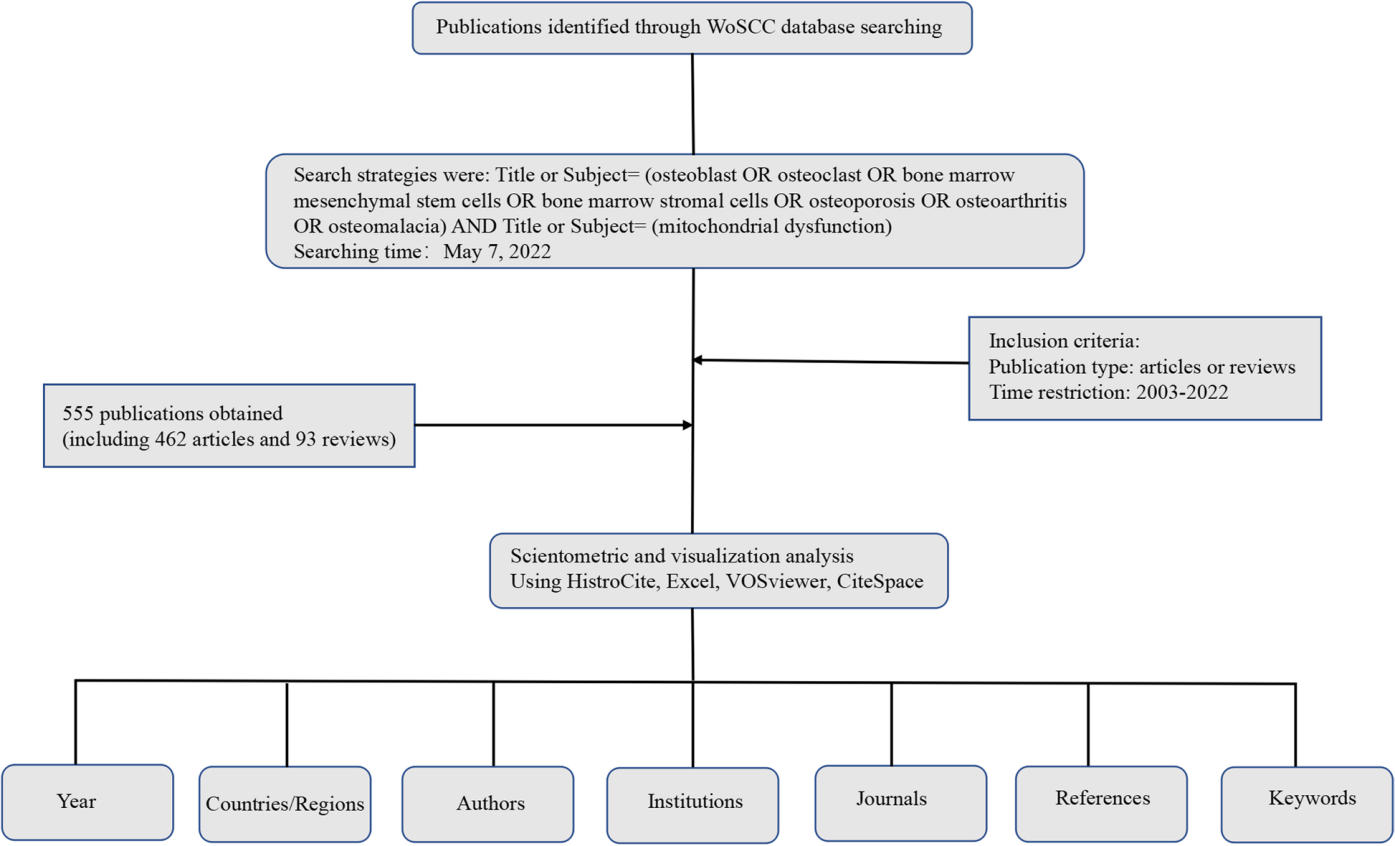
Flow diagram of literature search, screening, and analysis

WoSCC of Southern Medical University was searched on May 7, 2022. Because there are few literatures in this field, we collated all relevant studies on the impact of mitochondrial dysfunction on bone metabolism and related diseases published before the search time. Database sources are limited to SCIE and paper types are articles or reviews. The search strategies were: Title or Subject = (osteoblast OR osteoclast OR bone marrow mesenchymal stem cells OR bone marrow stromal cells OR osteoporosis OR osteoarthritis OR osteomalacia) AND Title or Subject = (mitochondrial dysfunction). To guarantee the accuracy of the data and obtain more useful information, on May 7, 2022, we downloaded all appropriate statistics from WoSCC and used the scientific metrology tool for additional investigation (Fig. 1).

## Statistical analysis

The annual production (amount of articles), publishing languages, and publishing types were determined using HistroCite (12.03.07) [21]. Information on IFs was searched in the Journal Citation Reports (JCR). VOSviewer (1.6.16) was selected to identify active countries/regions, journals, scholars, and most often cited journals, scholars, and citations, and relevant visual relationships [17, 24]. We also used Scimago Graphica to create a distribution network for related publications. Countries/regions, institutions, and publications are all represented by different nodes on the VOSviewer connection maps. The nodes represent the count of studies or co-occurrence frequencies. The co-occurrence associations are represented by the links between nodes, and the size of the linkages shows the co-occurrence frequencies of the two nodes [19]. The following VOSviewer reader settings were used: counting technique (full counting), whereas thresholds (T) of elements (countries/regions, institutions, journals, authors, and references) were used according to particular scenarios. CiteSpace (6.1.R2) [25] investigates the trends and noteworthy scientific works in a specific research direction [26, 27], and we used it to create a co-occurrence graph of keywords and then to cluster all the keywords. We used Microsoft Office Excel 2021 to manage the data and examine the publication trend (Fig. 1).

## Results

### Countries/regions

**Fig. 2:**
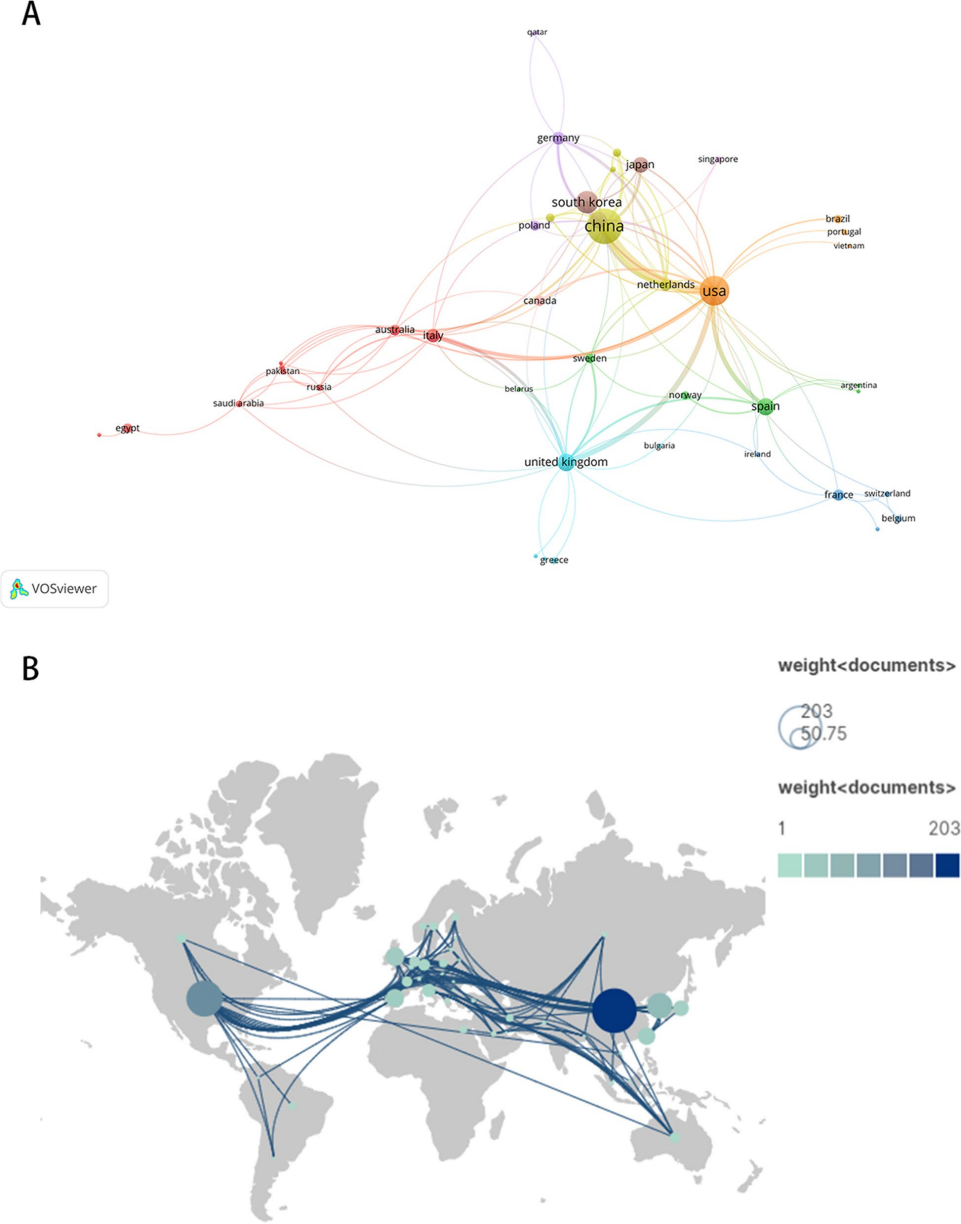
The proportion (**A**) and country network diagram (**B**, T = 1) associated with this research

As Supplementary Table 1 shows, 48 countries/regions collaborated on 555 articles in total. In the top 10 countries/regions in terms of production, six were less than 35 articles: Spain (*n* = 33), Taiwan (*n* = 31), Japan (*n* = 25), Italy (*n* = 18), Germany (*n* = 14) and Netherlands (*n* = 12). The remainders were more than 35, with China producing the most papers (*n* = 203), the United States ranked second (n = 133), then followed by South Korea (*n* = 66), the United Kingdom (*n* = 36). China, the United States, South Korea, and the United Kingdom have larger node sizes in this network map for more publications. But in terms of citions, the United States having the most citions (*n* = 7795), China ranked second (*n* = 4316), then followed by Spain (*n* = 1500). There was a lot of beneficial cooperation between different countries/regions (Fig. 2), which should be noticed.

### Authors and co-cited authors

**Fig. 3:**
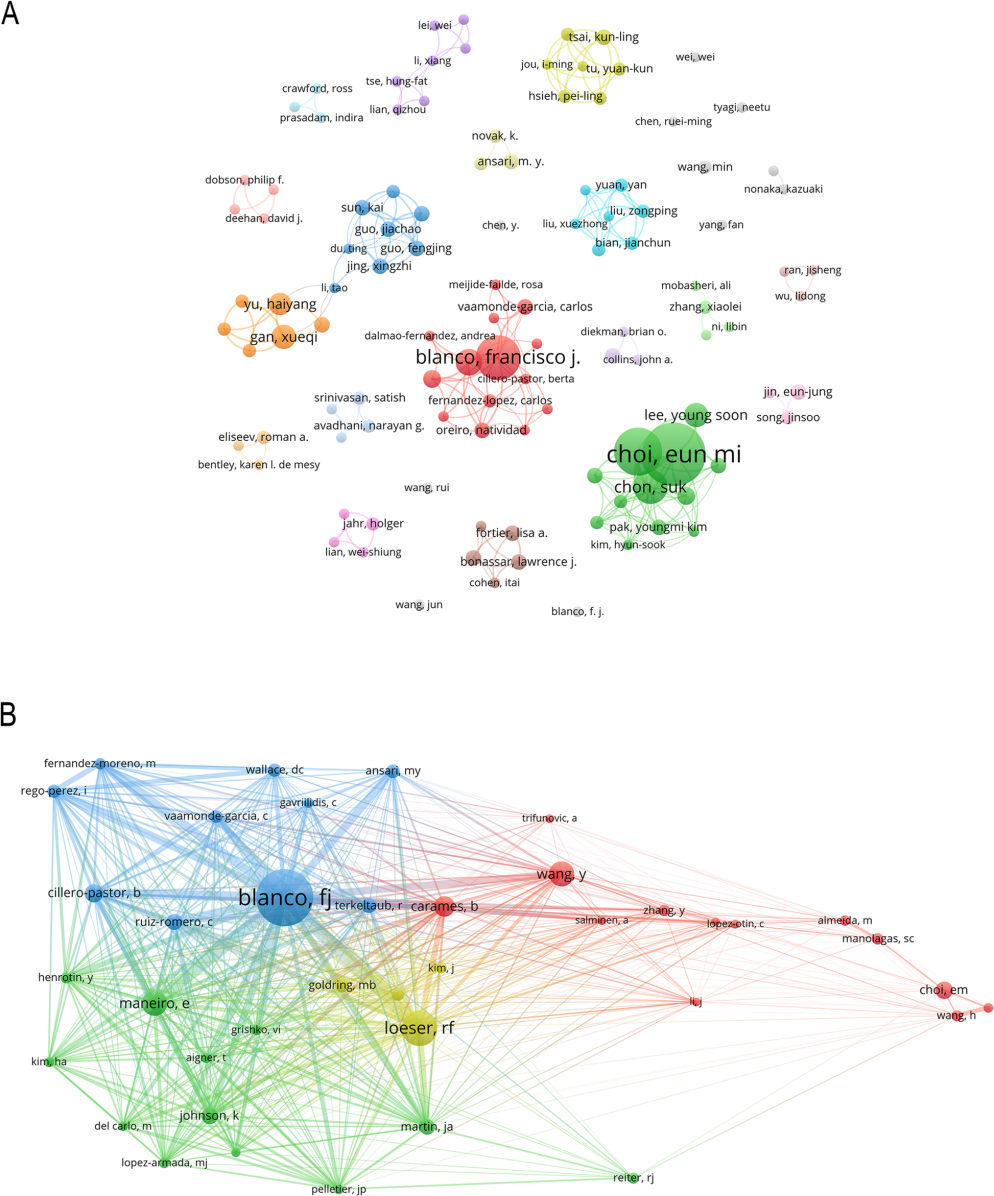
The authors of this study (**A**, T = 3) and co-cited authors (**B**, T = 30) are shown in the network map

This linked study involved a total of 3,109 authors. Ten authors contributed to the publication of five papers. Choi EM rated top and published the most papers (*n* = 32), followed by Suh KS (*n* = 22) and Blanco FJ (*n* = 20). Chon S came in fourth place (*n* = 14). Six to ten publications were published by the remaining four authors (Table 1). The co-authorship map was constructed using the authors (109/3,109, 0.75 percent) with a publication number greater than or equal to 3 (T = 3) (Fig. 3A). Because they have more publications, Choi EM, and Blanco FJ have higher node sizes. Several authors, including Choi EM and Suh KS, Blanco FJ, and others worked closely together.

**Table 1:** The top 10 authors and co-cited authors related to this field

Rank	Author	Count	Co-cited author	Co-citation
1	Choi EM	32	Blanco FJ	198
2	Suh KS	22	Loeser RF	123
3	Blanco FJ	20	Maneiro E	87
4	Chon S	14	Wang Y	87
5	Rego-Perez I	10	Carames B	72
6	Lee YS	9	Cillero-Pastor B	63
7	Gan XQ	9	Choi EM	61
8	Yu HY	8	Johnson K	57
9	Fernandez-Moreno M	6	Ruiz-Romero C	56
10	Rhee SY	6	Martin JA	55

Writers who have been cited in numerous publications are referred to as co-cited authors [27]. Two authors had more than 80 co-citations among the 21,149 co-cited authors. Blanco FJ rated top-1 with the most co-citations (*n* = 198), followed by Loser RF (*n* = 123), Maneiro E (*n* = 87), and Wang Y (*n* = 87) (Table 2). The remaining six top authors received 55 to 72 co-citations. The co-citation map was created using writers (39/21,149, 0.75%) who had at least 30 co-citations (T = 30) (Fig. 3B). As shown in Fig. 3B, Blanco FJ has the most co-citations with the biggest node area and dynamic co-cited relationships with Maneiro E, Cillero-Pastor B, Wang Y, and others.

**Table 2:** The top 15 journals and co-cited journals related to this field

Rank	Journal	Count	IF2022	Q	Journal	Co-citation	IF2022	Q
1	Osteoarthritis and Cartilage (England)	19	6.576	Q1	Journal of Biological Chemistry(United States)	916	5.486	Q1
2	Scientific Reports (England)	16	4.996	Q1	Osteoarthritis and Cartilage(England)	902	6.576	Q1
3	International Journal of Molecular Sciences (United States)	13	6.208	Q2	Arthritis and Rheumatism(United States)	611	10.995	Q1
4	Journal of Cellular Physiology (United States)	10	6.513	Q2	Nature(England)	575	69.504	Q1
5	Plos One(United States)	10	3.752	Q2	Proceedings of The National Academy of Sciences(United States)	566	12.779	Q1
6	Biochemical and Biophysical Research Communications (United States)	9	3.322	Q2	Plos One(United States)	530	3.752	Q2
7	Stem Cell Research & Therapy (England)	9	8.079	Q2	Free Radical Biology and Medicine(United States)	456	8.101	Q1
8	Cell Death & Disease (England)	8	9.685	Q1	Cell(United States)	432	66.850	Q1
9	Journal of Orthopaedic Research (England)	8	3.102	Q1	Science(United States)	407	63.714	Q1
10	Oxidative Medicine and Cellular Longevity (United States)	8	7.310	Q2	Annals of The Rheumatic Diseases (England)	399	27.973	Q1
11	Bone(United States)	7	4.626	Q1	Journal of Bone and Mineral Research (United States)	358	6.390	Q1
12	Molecular Medicine Reports (Greece)	7	3.423	Q4	Biochemical and Biophysical Research Communications(United States)	351	3.322	Q3
13	Biomedicine & Pharmacotherapy (France)	6	7.419	Q2	Bone(United States)	317	4.626	Q2
14	Faseb Journal (United States)	6	5.834	Q1	Arthritis Research & Therapy(England)	303	5.606	Q2
15	Free Radical Biology And Medicine(United States)	6	8.101	Q1	Nature Reviews Rheumatology(United States)	302	32.286	Q1

### Co-cited academic journals

**Fig. 4:**
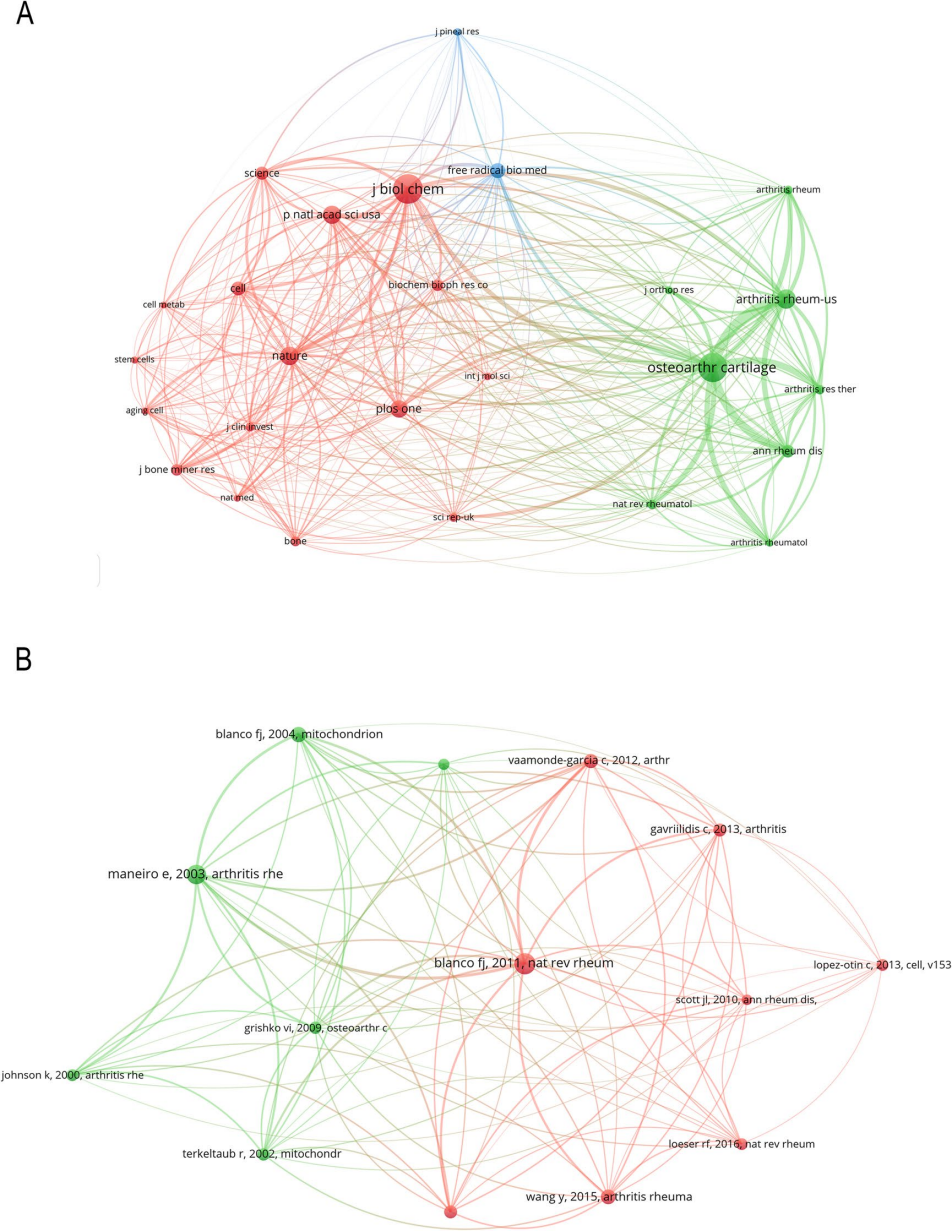
The network map of co-cited academic journals (**A**, T = 200) in this study; the co-cited network map of papers in this field (**B**, T = 25)

Of the 3,464 academic journals cited together, six had more than 500 co-citations, four of them are from the United States and the remaining two are from the United Kingdom (Table 2). The Journal of Biochemistry had the most co-citations (*n* = 916, IF2022 = 5.486, Q1), followed by Osteoarthr and Cartilage (*n* = 902, IF2022 = 6.576, Q1), Arthritis (*n* = 611, IF2022 = 10.995, Q1), Nature (*n* = 575, IF2022 = 69.504, Q1), Proceedings of The National Academy of Sciences (*n* = 566, IF2022 = 12.779, Q1). Among the top 15 journals cited jointly, Nature had the highest IF of 69.504 in 2022. Periodicals with joint citations greater than or equal to 200 (T ≥ 200) (26/3,464, 0.75%) were used to build collaborative citation networks (Fig. 4A) (Table 2). In that network, the Journal of Biochemistry has a larger node size due to more citations. Biochemistry has a positive co-citation with Nature and Science, and Skeletal Muscle Cartilage has a positive co-citation with Arthritis.

### Co-cited references

Co-Cited References are those that are quoted jointly by other papers [28]. There were 27,741 co-cited references across 555 connected publications showing the top ten references that were co-cited. Ten references were cited at least 31 times, and five were cited more than 40 times. The co-cited reference map was created using papers (14/27,741, 0.05%) who had at least 25 co-citations (T = 25) (Fig. 4B) (Supplementary Table 2).

As the supplementary table 3 shows, with 78 co-citations, Blanco FJ et al. published the top-1 co-cited paper in 2011. This research looked at the involvement of mitochondria in osteoarthritis [29]. Maneiro E et al. published the top-2 co-cited papers in Arthritis Rheum. They studied the activities of the respiratory chain enzyme complex and citrate synthase (CS), as well as changes in mitochondrial membrane potential, and discovered that mitochondrial function is involved in the pathophysiology of OA; cartilage degradation by OA and cartilage aging may be two distinct processes [30]. In 2004, Blanco FJ et al. published the top-3 co-cited publication in Mitochondrion [31]. In this research, the role of mitochondria in OA disease has been analyzed actually, and there is some evidence that suggests a possible relation between the dysfunction of mitochondria in chondrocytes and cartilage degradation. Arthritis Rheumatol published the top-4 co-cited Analytical study by Wang Y et al. in 2015 [32]. Mitochondrial biogenesis is poor in human OA chondrocytes, and this defect enhances chondrocyte precatatonic responses, according to this study. They also discovered that TFAM-mediated activation of the AMPK/SIRT-1/PGC-1 pathway reverses these effects, implying that pharmacologic AMPK activators have the potential to restrict OA progression. The top-5 co-cited reference was published in 2012 by Vaamonde-García C et al. [33]; it indicated that mitochondrial dysfunction could amplify the responsiveness to cytokine-induced chondrocyte inflammation through ROS production and NF-κB activation and that pathway might lead to the impairment of cartilage and joint function in OA. Gavriilidis C et al. published the top-6 co-cited study in 2013 [34]. This is the first study to evaluate the impact of SOD2 depletion on oxidative and mitochondrial function in human articular chondrocytes. The findings reveal that SOD2 deficiency in chondrocytes causes oxidative damage and mitochondrial dysfunction, implying that SOD2 downregulation may play a role in the etiology of OA. Ruiz-Romero C et al. published the top-7 most often cited publication in Mol Cell Proteomics in 2009 [35]. This work compares the mitochondrial protein profiles of normal and OA chondrocytes, indicating that mitochondrial dysregulation occurs in chondrocytes during OA and emphasizing redox imbalance as a crucial component in the pathogenesis of OA. Terkeltaub, R et al. reviewed several potential mechanisms by which NO leads to Chondrocyte damage and published the top-8 co-cited paper [36]. This contributes to a better understanding of the NO-related mechanisms that Cause chondrocyte damage and may lead to the happening of OA. Grishko VI et al. produced the top-9 most widely co-cited publication in 2009 in Nature Genetics [37]. They extracted chondrocytes from the knee joints of the remains of OA patients and then examined the integrity and repair ability of mtDNA, and found that mtDNA damage and mtDNA repair ability were poor, and that elimination of oxidative stress-induced damage may contribute to the pathogenesis of OA. The top-10 co-cited reference came from a review published in the Arthritis Rheum in 2000 by Johnson K et al. [38]. This research found that NO regulates mitochondrial ATP production in chondrocytes, and NO-induced inhibition of chondrocyte respiration regulates matrix loss and secondary cartilage mineralization in OA.

### Annual growth trend of publications

**Fig. 5:**
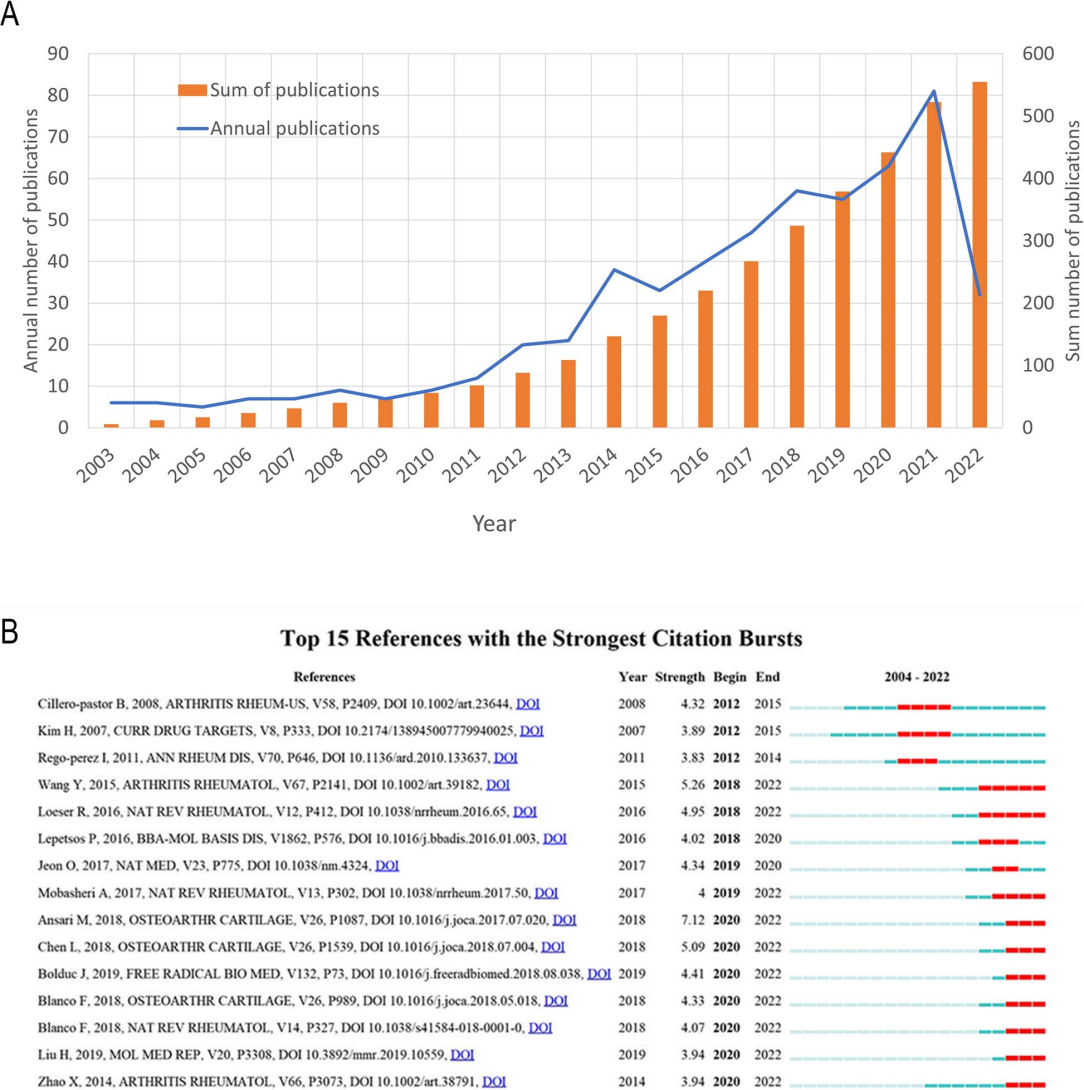
Flow diagram of literature search, screening, and analysis (**A**). Top 15 publications with a plenty of citations. References that were commonly cited are depicted by red bars, whereas references that were rarely cited are represented by blue bars (**B**)

Between 2003 and 2022, we found 555 linked studies, comprising 462 papers and 93 reviews. The annual production of linked research from 2003 to 2022 is growing almost all the time, as illustrated in Fig. 5A. The fewest papers were published in 2005 (5, 0.9%), and more than 40 papers were produced each year in the previous five years, with an average of roughly 28 papers published per year in the last twenty years. Since 2018, more than 50 papers have been published each year, peaking in 2021 (*n* = 81, 15%). A total of 32 papers were published in the first four months of 2022 and 77 papers are expected to be published in 2022.

### Citation burstness of references

Citation burst describes references that have been frequently and intently studied by academics on a particular subject at a particular time [20]. CiteSpace set the minimum period of the burst for associated articles to five years, and 15 references were discovered with substantial citation bursts. In Fig. 5B, each red or blue bar represents a time period, and a singular bar equals one year. The citation burst is highlighted in red [20]. Approximately 73.33% (11/15) of the references experienced a citation burst between 2015 and 2019.

Ansari MY et al. published the first paper, with the strongest burst strength (*n* = 7.12) in 2017 [39], and the citation burst lasted three years (2020–2022). The first paper determined the expression and role of Parkin in the clearance of damaged/dysfunctional mitochondria, regulation of reactive oxygen species (ROS) levels, and chondrocyte survival under pathological conditions. Their findings show that Parkin works to eradicate depolarized/damaged mitochondria in chondrocytes, which is required for mitochondrial quality control, ROS reduction, and chondrocyte survival under pathological settings. Wang Y et al. described that mitochondrial dysfunction is functionally defective in human chondrocytes and that the pathway in which APMK is located inhibits chondrocyte breakdown [32]. The study was published in Arthritis Rheumatol in 2015, with the second-highest citation burst (*n* = 5.26) lasting five years (2018–2022). The third-highest citation burst was written in 2018 by Chen LY et al. and published in Osteoarthritis Cartilage. had a burst strength of 5.09 with the burst lasting for three years (2020–2022) [40]. AMPK activation, via SIRT3, limits oxidative stress and improves mtDNA integrity and function in OA chondrocytes. These effects likely contribute to the chondroprotective effects of AMPK activity. This paper exhibited the fourth-highest citation burst by Loeser RF et al. in 2016 [41]. In his review, this author identifies mitochondrial dysfunction and oxidative stress as one of the potential mechanisms of aging leading to OA. This paper had a burst strength of 4.95. Finally, Bolduc JA et al. published the article with the fifth-highest citation burst (*n* = 4.41) in Free Radical Biology and Medicine in 2019 [42], and it lasted from 2020 until 2022. In his review, this author shows that peroxides accumulate with age and may damage chondrocytes either directly by damaging their structure or indirectly by affecting cellular conduction pathways.

Overall, the top 15 references' burst strength ranged from 3.83 to 7.12, while endurance strength ranged from 2 to 4 years.

### Keywords

**Fig. 6:**
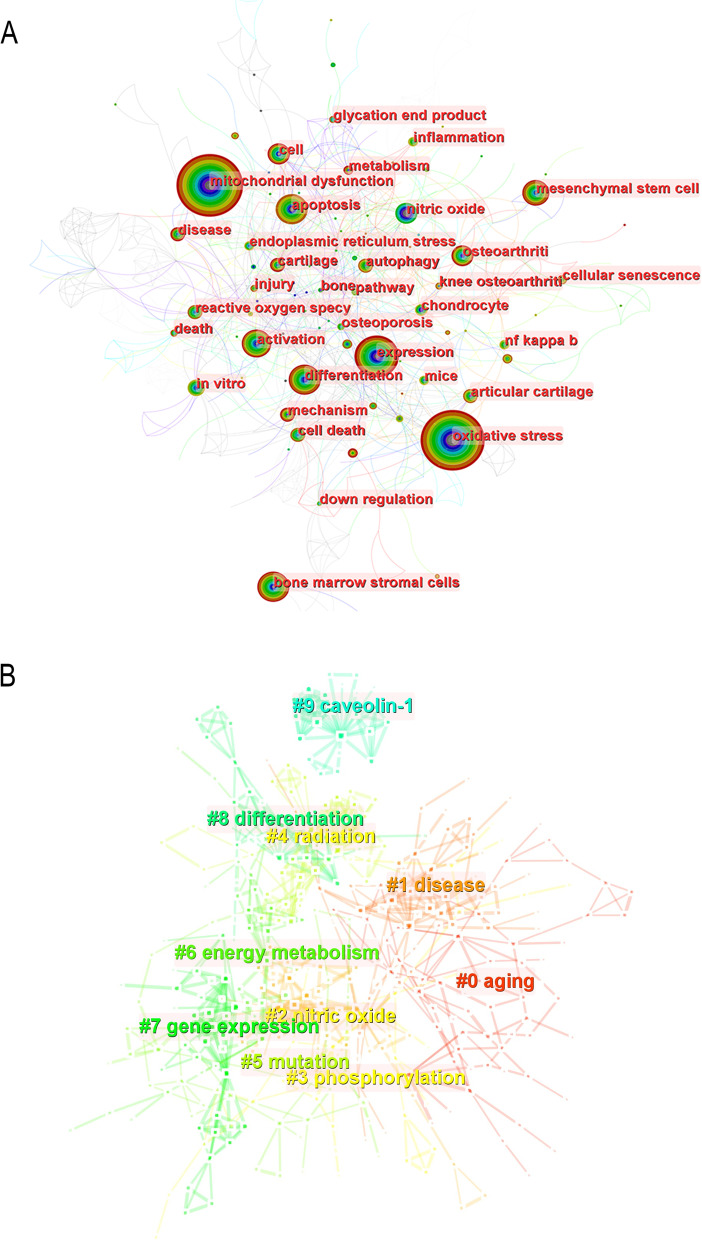
Keywords co-occurrence (**A**) and keywords aggregation analysis (**B**)

CiteSpace software was used for keywords and cluster analysis, and a total of 507 keywords and 19 clusters were obtained. The top 10 keywords were all no less than 55 times, in order of frequency, they are mitochondrial dysfunction, oxidative stress, expression, apoptosis, bone marrow stromal cells, differentiation, mesenchymal stem cell, nitric oxide, osteoarthritis, and activation (Supplementary Table 3). Seven keywords were published over 50 times. Mitochondrial dysfunction published the most times (*n* = 210) and rated top-1, the next were oxidative stress (*n* = 182) and expression (*n* = 90). The remaining four keywords were published 52 to 72 times. The keywords were used to construct the relationship map using CiteSpace (Fig. 6A). Because of the greater number of articles, the node sizes of mitochondrial dysfunction and oxidative stress are larger. Several themes, such as expression and cell death, mitochondrial malfunction and osteoporosis, and so on, collaborated closely.

According to the number of keywords included, all keywords can be divided into 19 clusters: aging, disease, nitric oxide, phosphorylation, radiation, mutation, energy metabolism, gene expression, differentiation, caveolin-1, oxidative stress, reactive oxygen species, advanced glycation end products, skeletal muscle, mitochondrial dysfunction, pathophysiology, oxidative stress, osteoblasts, mitochondrial function. Among nineteen clusters, six clusters had co-citations over 30. Cluster 1 “aging” had the most publications (*n* = 58) and rated the top-1, the next were “disease” (*n* = 47), “nitric oxide” (*n* = 39), and “phosphorylation” (*n* = 36). The remaining three clusters had publications from 33 to 36. The cluster picture was built using the top 10 clusters with the most terms. Figure 6B shows that "aging" has the largest size and the most frequency of occurrence.

## Discussion

### Annual growth trend analysis

In the past 20 years, 743 institutions from 49 countries/territories published 555 papers in 274 peer-reviewed journals, with 28,301 references in two languages. The trend from 2003 to 2021 predicts that nearly 100 studies will be published in 2022, suggesting a rise in interest in publications related to bone metabolism and mitochondrial dysfunction, particularly over the past ten years. As shown in the Fig. 5, before 2010, we can observe that there were less than 10 relevant publications per year, and there was no significant growth trend. However, in 2012–2021, the related literature almost showed an explosive growth. In 2021, there are 81 publications, which is more than the total number of publications from 2003 to 2011. This reflects the fact that related research has become a hot topic in recent years.

### Countries/regions analysis

The first ten countries/regions are spread over three areas: Asia, America, and Europe, with Asia accounting for half of them (Fig. 2). For the reason why research on mitochondrial dysfunction on bone metabolism and related diseases has been concentrated in Asia, we speculate that there may be increasing emphasis on the study of bone metabolic diseases in Asiain countries and regions in the last 20 years as mentioned in the rencent study [43]. As Supplementary table 1 shows, the United States (*n* = 133) has about two-thirds as many publications as China (*n* = 203) does overall, but has almost twice (*n* = 7795) as many citations, demonstrating the superior caliber of American literature in the field (Supplementary Fig. 1). Furthermore, since only China is a developing country among the top 10 countries/regions in this field, developing countries' research capacities in this area need to be strengthened. As a result, developing nations should actively absorb knowledge from developed nations and contribute concepts to advance this research.

### Author analysis

Of the authors, Choi EM published most of the research, with Blanco FJ cited the most. In addition, we found that Choi EM and Blanco FJ are the first 10 prolific authors, as well as the first 10 co-cited authors, suggesting that they have a significant contribution in this area. Blanco FJ, in particular, published more works and gained more citations, implying that his team may be good potential collaborators in this field.

### Institution and journal analysis

As the Supplementary Table 4 and supplement Fig. 2A shows, eight of the 10 most prolific institutions in the field came from China, the other two from South Korea, and the most prolific was Kyung Hee University. However, it is worth noting that of the top 10 institutions considered to have more influence in this area, nine are located in Europe and the United States, with the most cited institution being the University of North Carolina in the United States. Of the top 5 most cited institutions, only Kyung Hee University is from Asia (Supplementary Fig. 2B). Scholars should pay more attention to these institutions when conducting research in this area and could develop cooperative relationships with them. Osteoarthritis and Cartilage have been published in most studies, and biochemistry is the most commonly cited journal. At the same time, American journals have the largest proportion of the first 10 journals and co-cited journals (50 and 73.3%, respectively), suggesting that American journals contribute to many studies and generate a lot of interest. Furthermore, we discovered more co-citations in journals with high synthesis factors, indicating that these journals are regularly co-cited and play a key role in this field. When we write, we can refer to the most cited academic journals, and when we submit, we can consider productive academic journals.

### Co-cited publications and citation burstiness analysis

Co-cited references show the frequency with which two papers are referenced by other papers; thus, they could be seen in a certain field as a knowledge base [23]. The top ten co-cited references in this scientometric review were chosen to determine the knowledge base on the effects of mitochondrial dysfunction on bone metabolism and related illnesses.

In general, the top 10 co-citations were about the following topics: Pathogenesis, related pathways and proteins, related diseases, and therapeutic drugs, all of these were the foundations for research in this field.

Ten publications' citation burstiness ended in 2022 or later. They represented the field's new emergent hosts and were recommended for further study.

### Keywords analysis

Keywords represent the basic research directions in this field. For example, Trifunovic, A's experimental results suggest that increasing mammalian age may lead to the accumulation of mitochondrial DNA (mtDNA) mutations in somatic cells and a decrease in respiratory chain function, then causing a range of diseases, including osteoporosis [44]. Lepetsos, P suggests that new strategies for the treatment of osteoarthritis can be explored in terms of signaling pathways related to mitochondrial oxidative stress [45]. Zaman, G found that the expression of both basal and load-related genes in resident bone cells is significantly influenced by the environment by probing the expression of genes related to bone metabolism [46]. 19 clusters can be broadly classified into three categories, the first on mechanisms of mitochondrial dysfunction, the second on interventional factors imposed in the study, and the third on cells and diseases related to bone metabolism. As Supplementary Table 3 shows, from the frequency of keywords, we can also find that the cells that are highly relevant to bone metabolism are bone marrow stromal cells (*n* = 69), Mesenchymal stem cells (*n* = 52) and Osteoarthritis (*n* = 50) is the most common bone metabolic disease. Meanwhile, in the keyword analysis, we may have missed some important information, such as the influence of skeletal muscle in this process of mitochondrial dysfunction on bone metabolism [47, 48], and future studies should pay more attention to this aspect.

## Strengths and limitations

There are numerous distinct advantages to our study. First, a systematic analysis of publications related to Mitochondrial dysfunction on bone metabolism and related diseases using science-based methods for the first time provides comprehensive guidance for clinicians and scholars working in this area.

Second, we conducted this study using four scientometric tools simultaneously, HistCite, VOSviewer, as well as CiteSpace are the most popular in scientific measurement. Therefore, there may be an objective data analysis process. Third, compared to conventional narrative assessments, the scientometric analysis offers a more accurate picture of how research trends and focuses have changed over time. Our study, like other scientific-metrological studies, has limitations. First, search for data only from WoSCC, without other databases for a supplement. However, we should know that WoSCC is the most comprehensive and reliable database. In addition, current scientometric software has difficulties when analyzing data from different databases at once. Second, all information is obtained using scientometric software, not hand-written in meta-analyses or systematic reviews. As a result, our results could be skewed. For example, the author's namesake cannot be ruled out, and the full information of the article cannot be accurately obtained by the software. We will discover better solutions owing to developments in machine learning, language processing, and information science. Finally, due to the paucity of literature published in this field, the total number of documents in our country is only 555.

## Conclusion

The study of the consequences of mitochondrial dysfunction on bone metabolism and associated diseases is advancing rapidly, and the related literature has shown explosive growth in the last few years. Countries represented by China have conducted in-depth research and strengthened cooperation in this field. Several prominent researchers like Blanco FJ and Sun L have published extensive literature on the subject and are widely cited. Future research in this area will focus on the factors leading to mitochondrial dysfunction, including oxidative stress, aging, gene expression, and the pathogenesis of bone metabolism-related diseases. The clinical implications of the current scientometric analysis could help us explore some potential treatment directions such as regulating inflammatory, catabolic factors, and mitochondrial transplantation for bone metabolic diseases [49].

## Supplementary Information

**Additional file 1:** **SupplementaryFigure 1.** The top 10 cited countrys related to this field.

**Additional file 2: SupplementaryFigure 2. **The network map of top 10 institutions (A) and the top 10 cited networkmap of institutions in this field (B).

**Additional file 3:** **Supplementarytable 1.** The top 10 country and cited country in this field.

**Additional file 4:** **SupplementaryTable 2.** The top 10 co-cited references related to this field

**Additional file 5: Supplementary Table 3. **The top 10 Keywordsrelated to this field.

**Additional file 6: Supplementary Table 4. **The top 10 institutions and cited institutions related to this field.

## Data Availability

All Authors had full access to all study data and take full responsibility for the accuracy of the data analysis. The data that supports the fnding of this study are available from the corresponding author upon request.
