# Supplementary figures and images for: Effects of mitochondrial dysfunction on bone metabolism and related diseases: a scientometric study from 2003 to 2022
Source: BMC Musculoskelet Disord. 2022 Nov 26;23:1016. doi: 10.1186/s12891-022-05911-8 (PMC9701404; doi:10.1186/s12891-022-05911-8)

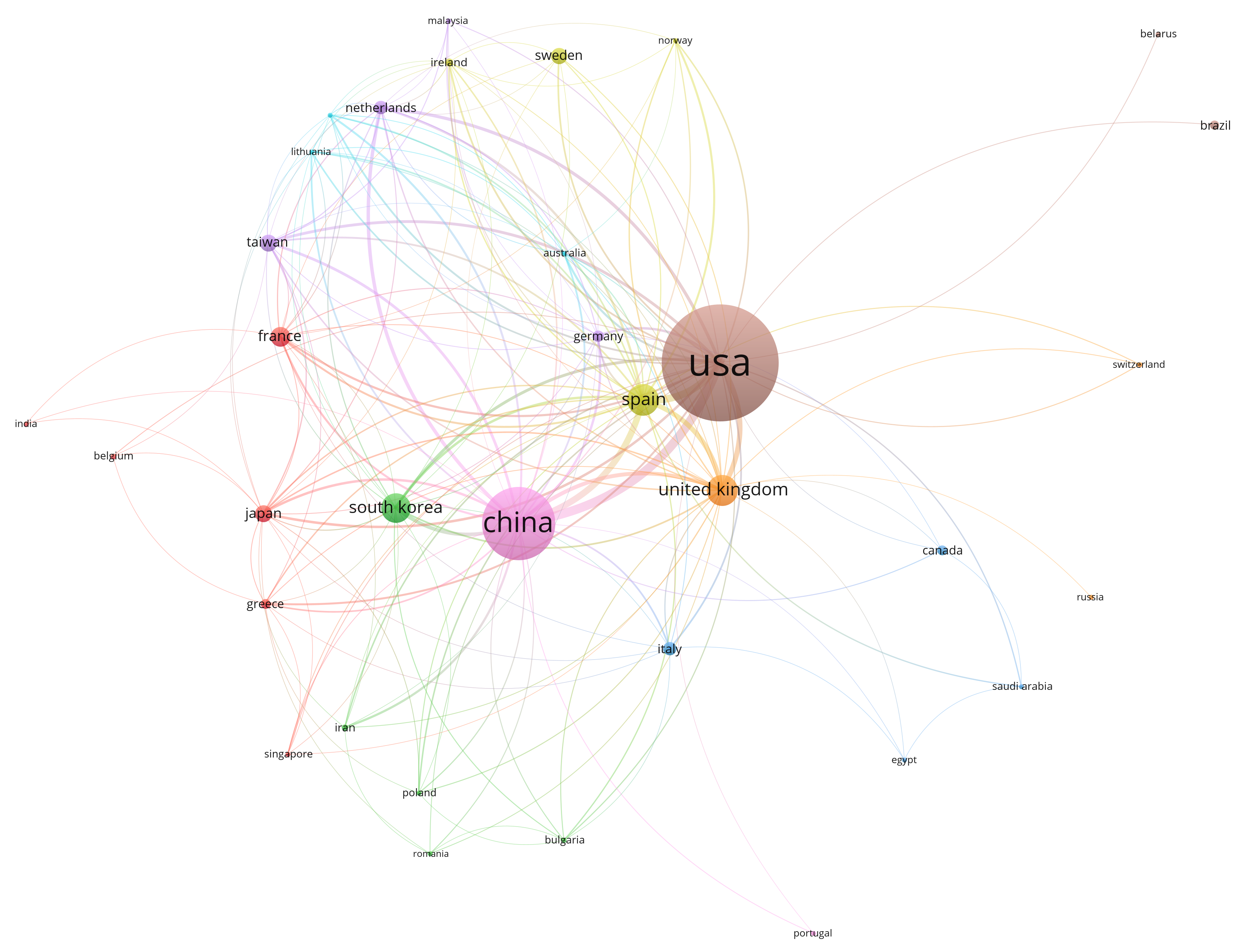

Supplement: Supplementary file 1 — Additional file 1: SupplementaryFigure 1. The top 10 cited countrys related to this field. [file 12891_2022_5911_MOESM1_ESM.tiff]

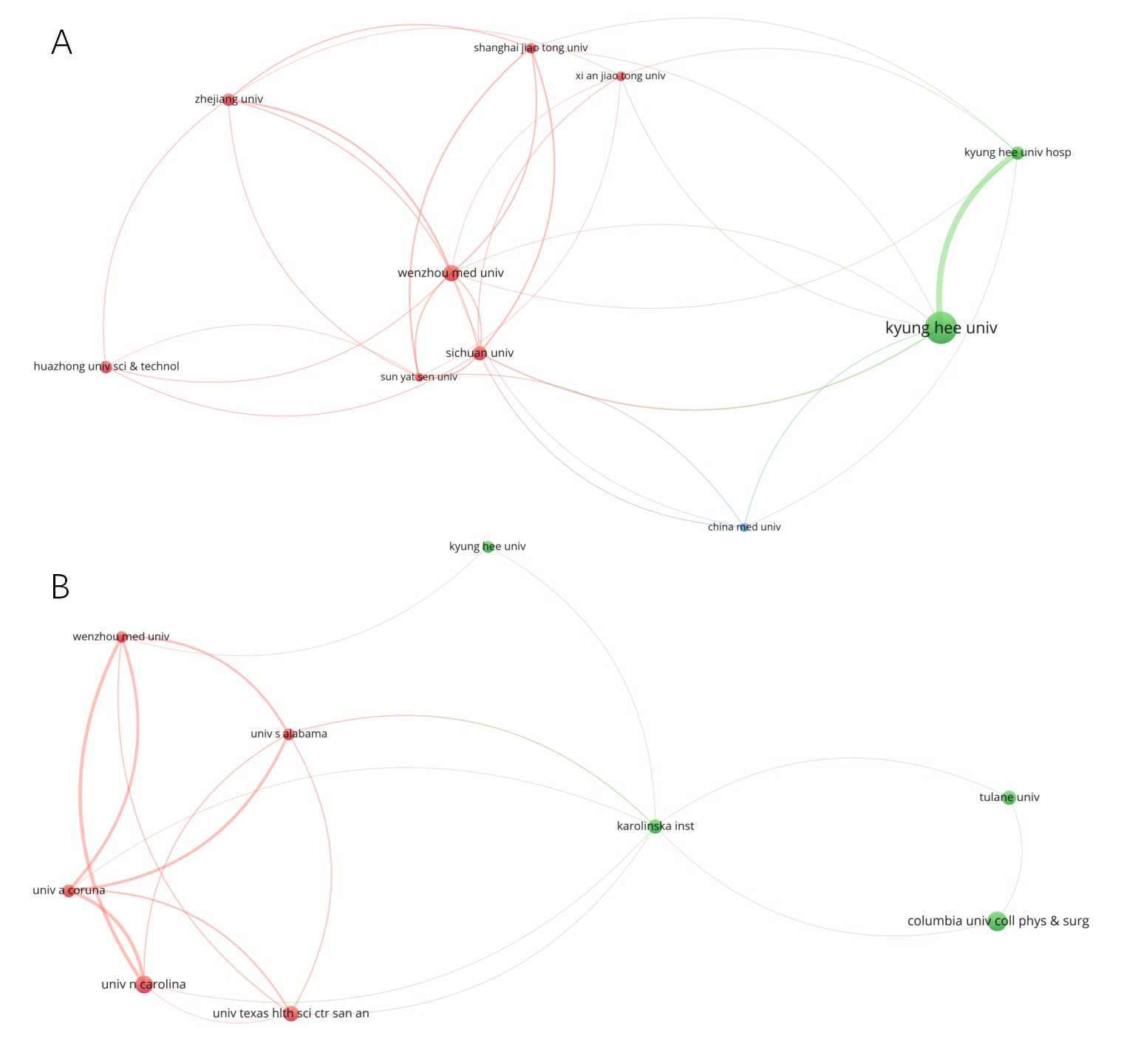

Supplement: Supplementary file 2 — Additional file 2: SupplementaryFigure 2. The network map of top 10 institutions (A) and the top 10 cited networkmap of institutions in this field (B). [file 12891_2022_5911_MOESM2_ESM.tif]
