# Supplementary material for: Effects of mitochondrial dysfunction on bone metabolism and related diseases: a scientometric study from 2003 to 2022
Source: BMC Musculoskelet Disord. 2022 Nov 26;23:1016. doi: 10.1186/s12891-022-05911-8 (PMC9701404; doi:10.1186/s12891-022-05911-8)
Supplement: Supplementary file 3 — Additional file 3: Supplementarytable 1. The top 10 country and cited country in this field. [file 12891_2022_5911_MOESM3_ESM.docx]

Supplementary table 1 The top 10 country and cited country in this field

| Rank | Country/Region | Documents | Country/Region | Citations |
| --- | --- | --- | --- | --- |
| 1 | China | 203 | The United States | 7795 |
| 2 | The United States | 133 | China | 4316 |
| 3 | South Korea | 66 | Spain | 1500 |
| 5 | The United Kingdom | 36 | South Korea | 1433 |
| 4 | Spain | 33 | The United Kingdom | 1346 |
| 6 | Taiwan | 31 | France | 811 |
| 7 | Japan | 25 | Japan | 661 |
| 8 | Italy | 18 | Taiwan | 657 |
| 9 | Germany | 15 | Sweden | 637 |
| 10 | Netherlands | 12 | Italy | 488 |
