# Supplementary material for: Effects of mitochondrial dysfunction on bone metabolism and related diseases: a scientometric study from 2003 to 2022
Source: BMC Musculoskelet Disord. 2022 Nov 26;23:1016. doi: 10.1186/s12891-022-05911-8 (PMC9701404; doi:10.1186/s12891-022-05911-8)
Supplement: Supplementary file 4 — Additional file 4: SupplementaryTable 2. The top 10 co-cited references related to this field [file 12891_2022_5911_MOESM4_ESM.docx]

| Rank | Co-cited Reference | Count | Country |  |  |
| --- | --- | --- | --- | --- | --- |
| 1 | Blanco FJ, 2011, Nat Rev Rheumatol, V7, P161 | 78 | Spain |  |  |
| 2 | Maneiro E, 2003, Arthritis Rheum, V48, P700 | 66 | Spain |  |  |
| 3 | Blanco FJ, 2004, Mitochondrion, V4, P715 | 47 | Spain |  |  |
| 4 | Wang Y, 2015, Arthritis Rheumatol, V67, P2141 | 43 | The United States |  |  |
| 5 | Vaamonde-Garcia C, 2012, Arthritis Rheum-US, V64, P2927 | 41 | Spain |  |  |
| 6 | Gavriilidis C, 2013, Arthritis Rheum-US, V65, P378 | 36 | The United Kingdom |  |  |
| 7 | Ruiz-Romero C, 2009, Mol Cell Proteomics, V8, P172 | 36 | Spain |  |  |
| 8 | Terkeltaub R, 2002, Mitochondrion, V1, P301 | 33 | The United States |  |  |
| 9 | Grishko VI, 2009, Osteoarthr Cartilage, V17, P107 | 32 | The United States |  |  |
| 10 | Johnson K, 2000, Arthritis Rheum-US, V43, P1560 | 31 | The United States |  |  |

**S**upplementary Table 2 The top 10 co-cited references related to this field
