# Supplementary material for: Effects of mitochondrial dysfunction on bone metabolism and related diseases: a scientometric study from 2003 to 2022
Source: BMC Musculoskelet Disord. 2022 Nov 26;23:1016. doi: 10.1186/s12891-022-05911-8 (PMC9701404; doi:10.1186/s12891-022-05911-8)
Supplement: Supplementary file 5 — Additional file 5: Supplementary Table 3. The top 10 Keywordsrelated to this field. [file 12891_2022_5911_MOESM5_ESM.docx]

| Rank | Keywords | Count | Cluster number | Cluster name |
| --- | --- | --- | --- | --- |
| 1 | Mitochondrial dysfunction | 210 | 14 | Mitochondrial dysfunction |
| 2 | Oxidative stress | 182 | 10 | Oxidative stress |
| 3 | Expression | 90 | 7 | Gene expression |
| 4 | Apoptosis | 72 | 10 | Oxidative stress |
| 5 | Bone marrow stromal cells | 69 | 7 | Gene expression |
| 6 | Differentiation | 66 | 8 | Differentiation |
| 7 | Mesenchymal stem cell | 52 | 0 | Aging |
| 8 | Nitric oxide | 50 | 2 | Nitric oxide |
| 9 | Osteoarthritis | 50 | 5 | Mutation |
| 10 | Activation | 50 | 6 | Energy metabolism |

Supplementary Table 3 The top 10 Keywords related to this field
