# Supplementary material for: Effects of mitochondrial dysfunction on bone metabolism and related diseases: a scientometric study from 2003 to 2022
Source: BMC Musculoskelet Disord. 2022 Nov 26;23:1016. doi: 10.1186/s12891-022-05911-8 (PMC9701404; doi:10.1186/s12891-022-05911-8)
Supplement: Supplementary file 6 — Additional file 6: Supplementary Table 4. The top 10 institutions and cited institutions related to this field. [file 12891_2022_5911_MOESM6_ESM.docx]

| Rank | Institution | Documents | Institution | Citations |
| --- | --- | --- | --- | --- |
| 1 | Kyung Hee Univ | 39 | Univ N Carolina | 734 |
| 2 | Wenzhou Med Univ | 20 | Univ Texas Hlth Sci Ctr San Antonio | 655 |
| 3 | Sichuan Univ | 17 | Karolinska Inst | 588 |
| 4 | Kyung Hee Univ Hosp | 16 | Univ a Coruna | 544 |
| 5 | Huazhong Univ Sci & Technol | 15 | Kyung Hee Univ | 503 |
| 6 | ZheJiang Univ | 15 | Univ S Alabama | 494 |
| 7 | Shanghai Jiao Tong Univ | 13 | Wenzhou Med Univ | 484 |
| 8 | Xi An Jiao Tong Univ | 12 | Shanghai Jiao Tong Univ | 427 |
| 9 | Fourth Mil Med Univ | 11 | Xi An Jiao Tong Univ | 346 |
| 10 | China Med Univ | 11 | Taipei Med Univ | 345 |

supplementary table 4 : The top 10 institutions and cited institutions related to this field
